# The Distinct Role of HIF-1α and HIF-2α in Hypoxia and Angiogenesis

**DOI:** 10.3390/cells14090673

**Published:** 2025-05-04

**Authors:** Mouayad Zuheir Bakleh, Ayman Al Haj Zen

**Affiliations:** College of Health and Life Sciences, Hamad Bin Khalifa University, Doha P.O. Box 34110, Qatar

**Keywords:** hypoxia, HIF signaling, HIF switch, angiogenesis

## Abstract

Hypoxia results in a wide range of adaptive physiological responses, including metabolic reprogramming, erythropoiesis, and angiogenesis. The response to hypoxia at the cellular level is mainly regulated by hypoxia-inducible factors (HIFs): HIF1α and HIF2α isoforms. Although structurally similar and overlapping gene targets, both isoforms can exhibit distinct expression patterns and functions in some conditions of hypoxia. The interaction between these isoforms, known as the “HIF switch”, determines their coordinated function under varying oxygen levels and exposure time. In angiogenesis, HIF-1α is rapidly stabilized under acute hypoxia, prompting a metabolic shift from oxidative phosphorylation to glycolysis and initiating angiogenesis by activating endothelial cells and extracellular matrix remodeling. Conversely, HIF-2α regulates cell responses to chronic hypoxia by sustaining genes critical for vascular remodeling and maturation. The current review highlights the different roles and regulatory mechanisms of HIF-1α and HIF-2α isoforms, focusing on their involvement in cell metabolism and the multi-step process of angiogenesis. Tuning the specific targeting of HIF isoforms and finding the right therapeutic window is essential to obtaining the best therapeutic effect in diseases such as cancer and vascular ischemic diseases.

## 1. Introduction

Maintaining a balanced amount of cellular oxygen (O_2_) is crucial for the survival of eukaryotic organisms because oxygen is the primary substrate for generating ATP in the mitochondria, which is necessary for cell metabolic activities [1]. The expression of many genes related to cell adaptation and survival is altered upon exposing cells or organisms to low oxygen levels (hypoxia) [2]. Hypoxia-inducible factors (HIFs) have been recognized as the master molecular regulators of perceiving oxygen availability and controlling how cells respond to hypoxia. HIFs alter pathways that impact development, inflammation, angiogenesis, erythropoiesis, cell survival, apoptosis, and metabolism [3]. Their disturbance leads to the deregulation of O_2_ homeostasis, which is involved in a variety of pathologies, including tumorigenesis, vascular ischemic diseases, and immune inflammatory disorders. Both HIF-1α and HIF-2α isoforms have been demonstrated to be involved in adaptive responses to hypoxia [4]. Despite having similar structures and functional overlap, HIF-1α and HIF-2α have separate regulation systems and react differently to diverse stimuli [5]. In this review, we provide an overview of the different functional roles of HIF1 and HIF2 under hypoxia and their level switch in cell metabolism and angiogenesis.

## 2. HIF Isoforms’ Structure

**Figure 1:**
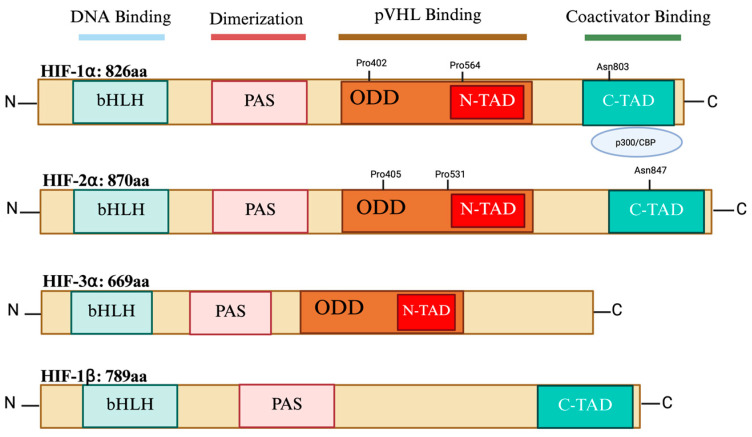
The structural composition of HIF-1α, HIF-2α, HIF-3α, and HIF-1β. bHLH and PAS domains interact with DNA and are involved in heterodimerization. The N-TAD and C-TAD are required for transcriptional activation, whereas the ODD is required for oxygen-dependent hydroxylation and degradation. ARNT: aryl hydrocarbon nuclear receptor translocator; bHLH: basic helix loop helix; PAS: Per-ARNT-Sim; ODD: oxygen-dependent degradation domain; N-TAD: N-terminal activation domain; C-TAD: C-terminal activation domain. The figure was created with BioRender.com.

HIFs are transcription factors that belong to the superfamily of basic helix–loop–helix (bHLH) proteins. They are heterodimers made up of an oxygen-regulated HIF-ααα subunit and a constitutively expressed HIF-β subunit also known as AHR nuclear translocator, ARNT [6]. Both the bHLH domain and per-ARNT-Sim (PAS) have been identified to be essential for HIF dimerization. However, the basic domain is involved in DNA binding, while the helix–loop–helix domain is involved in the dimerization between the two subunits. The PAS domain is involved in protein–protein interactions [7]. Both HIF-1α and HIF-2α isoforms contain other functional domains: terminal transactivation domains (TADs) and an oxygen-dependent degradation domain (ODD). However, HIF-3α does not have a C-TAD and acts as a dominant-negative regulator of HIF1/DNA binding [8]. The C-TAD interacts with coactivators like CBP/p300 to modulate the transcription of HIF-α in hypoxia, whereas the N-TAD is responsible for stabilizing HIF-α against degradation. The ODD is a highly conserved domain that overlaps the N-TADs and includes amino acids that are targets for hydroxylation, such as asparagine (N) and proline (P) residues under normal oxygen levels. Therefore, the ODD facilitates oxygen-regulated turnover and regulates the activity and stability of the alpha subunits [9] (Figure 1). The functional domains of the HIF-1α and HIF-2α isoforms share a significant degree of similarity and are subject to hydroxylation of particular proline and asparagine residues which controls both their expression and transcriptional activity [10]. HIF-1α and HIF-2α are identical in terms of amino acid sequence, at 48%, and in terms of their bHLH and PAS domains, they share 83% and 70% similarity, respectively [11].

All HIF-α isoforms can form a dimer with any HIF-β subunit (which has also three isoforms: 1β, 2β, and 3β) [12]. The heterodimer HIFα/HIFβ is required for binding to the HIF response element (HRE) on the DNA consensus sequence R/ACGTG (where R is A or G) in the promoters or enhancers of target genes [13]. HIF-1α-bound HRE motifs are mainly found in promoter neighboring regions, whereas HIF-2α binding tends to occur more in distal regions from the promoter site [14]. Distal and proximal hypoxia response elements cooperate in the case of regulation of organ-specific erythropoietin (EPO) gene expression. Also, it has been shown that more HRE motifs bind to HIF-1α in promoter open chromatin regions under acute hypoxia exposure (2–24 h), whereas more HRE motifs bind to HIF-2α under chronic hypoxia exposure (48–72 h) [15].

## 3. Oxygen-Dependent Regulation of HIF

HIF expression can constitutively occur independently of cellular oxygen levels. However, both HIF-1α and HIF-2α degrade rapidly under a normoxic environment (approximately between 5 and 8 min) [16]. Under normal oxygen levels, HIF regulatory proteins—the prolyl hydroxylase domain enzymes (PHD1, 2, and 3)—are activated [17,18] to maintain low HIF-α activity by hydroxylating two amino acids, proline (P) at P402 and P564 sites in HIF-1α or at P405/P531 sites in HIF-2α located in the LXXLAP amino acid motif in the ODD region [19,20]. While PHD enzymes are ubiquitously expressed, they display tissue-specific expression levels, with PHD2 being the most expressed and found in most types of tissue, whereas PHD1 is highly expressed in the testis and PHD3 is highly expressed in the heart [21]. In a cellular context, PHD enzymes display variable expression patterns. For example, PHD1 is predominantly expressed in the nucleus, while PHD2 is mainly found in the nucleus, and PHD3 appears in both [22]. The enzymatic activity of PHD is strictly dependent on the presence of α-ketoglutarate and Fe (II). This process is mediated by an iron-responsive element (IRE) located in the 5′ UTR region of HIF-α [23]. Therefore, PHD and FIH act as O_2_ sensors whereby they are only activated in the presence of consistent levels of molecular oxygen. This allows HIF-α to avoid proteolytic degradation and is translocated to the nucleus where it forms a transcription complex with HIF-β.

**Figure 2:**
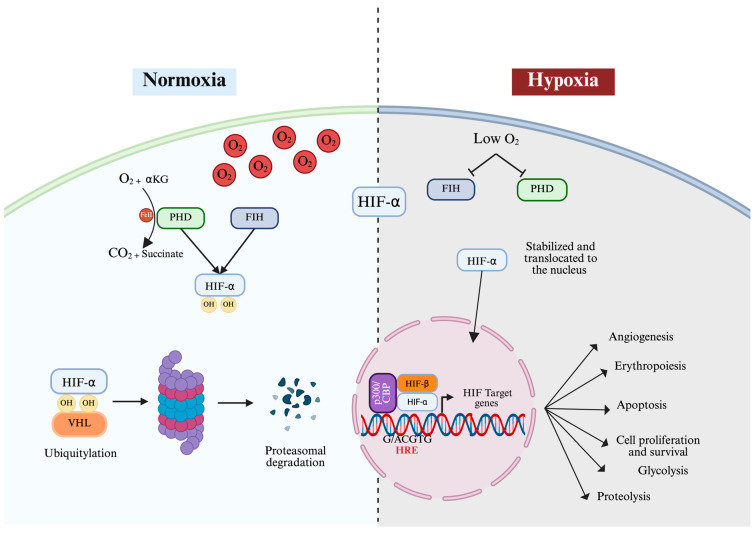
Canonical regulation of HIF-α activity under normal and hypoxic conditions. PHDs hydroxylate HIF-α on proline residues inside the ODD in the presence of oxygen (normoxia). Hydroxylation causes the Von Hippel–Lindau (VHL) E3 ligase complex to engage, ubiquitinating HIF-α and targeting it for proteasomal degradation. Hydroxylation of the asparagine residue through FIH precludes CBP/p300 from binding to HIF-α. PHD and FIH are inhibited and thereby the HIF-α subunit is stabilized due to the lack of hydroxylation. HIF-α is then translocated to the nucleus, where it dimerizes with HIF-β. The dimer binds to HREs and regulates target gene expression. The figure was created with BioRender.com.

Higher levels of oxygen cause the activation of an additional hydroxylase called the factor-inhibiting HIF (FIH), also known as asparaginyl hydroxylase, which causes the hydroxylation of asparagine (N803) of HIF-1α, leading to further inhibition of the p300/CReB (cAMP-response-element-binding protein) coactivator that usually binds the C-TAD of HIF-α and enhances its transcription [24]. Meanwhile, in HIF-2α, FIH causes the hydroxylation of asparagine (N847) and suppresses its activity [9,25]. The activity of FIH was found to be inhibited by oxidative stress caused by peroxides, therefore enhancing HIF activity even under normoxic conditions [26,27,28]. The hydroxylation of proline and asparaginyl molecules leads to HIF being tagged for ubiquitination and proteasomal degradation by a ubiquitin E3 ligase known as the Von Hippel–Lindau tumor suppressor protein (pVHL) [29]. The pVHL protein functions as the substrate recognition module of an E3 ubiquitin ligase complex called the ECR complex that includes cullin-2, ring-box 1, elongin C, and elongin B and regulates the polyubiquitylation and proteasomal degradation of HIF-α subunits [29]. The effects of PHD on various HIF isoforms are typically not similar as they exhibit varying affinities for HIF-α subunits, with PHD3 displaying greater affinity toward HIF-2α than HIF-1α, while PHD2 primarily targets HIF-1α [18] (Figure 2).

## 4. Oxygen-Independent Regulation of HIF

HIF-1α and HIF-2α can be regulated independently of cellular oxygen availability. This non-canonical regulation can occur at multiple levels: mRNA transcription, protein synthesis, or stabilization in response to other cellular stimuli such as inflammation and a nutrient-deprived environment [30]. These regulatory events frequently appear to be unique to a single HIF isoform or have opposing effects on the production of both isoforms: HIF-1α and HIF-2α [31].

It has been shown that pro-inflammatory and anti-inflammatory cytokines play an important role in the regulation of both HIF-1α and HIF-2α. For instance, IFN-γ released from Th1-lymphocytes can increase the stability and transcriptional activity of HIF-1α by inhibiting PHD enzyme activity [32,33]. Tumor Necrosis Factor α (TNF-α) stimulates the Nuclear Factor Kappa B (NF-κB) signaling pathway, leading to enhanced HIF-1α mRNA transcription [33]. Similarly, previous studies have shown that IL-1β can induce HIF-1α activity at several levels, including transcriptional and post-translational levels [34,35,36]. Stiehl et al. showed that the induction of HIF-1α expression is mediated by ERK and PI3K signaling activation [37]. IL-6 and IL-17 were also reported to cause an increase in the expression of HIF-1α through the STAT3 pathway [38,39]. In contrast, Th2-lymphocytes producing anti-inflammatory cytokine interleukin 4 (IL-4) activate HIF-2α mRNA transcription in M_2_ macrophages [40]. Another report showed that TGF-β can activate both HIF isoforms under normoxic conditions through the ALK5 signaling pathway [41]. FOXO3a, a transcription factor that plays a critical role in the inflammatory process, was reported to inhibit HIF-1α expression through the negative feedback activity of the transcriptional cofactor, CITED2 [42]. Furthermore, increased ROS levels, which are frequently associated with inflammation, can stabilize HIF-1α activity regardless of oxygen levels by activating the PI3K/AKT pathway [43].

Nutrient-altered conditions are also important for regulating HIF-1α and HIF-2α independently of oxygen levels. One report showed that cancer cells and endothelial cells supplemented with starvation medium caused an increase in the level of HIF-1α via cap-independent translation initiation [44]. Nishimoto et al. also reported that glucose deprivation increases HIF-1α levels and increases the DNA binding activity between HIF-1α, STAT3, and TCF4, causing overexpression of their downstream target genes [45]. In the same context, the increased amounts of fumarate and succinate can act as allosteric inhibitors and block PHDs, leading to HIF-1α stabilization [46]. The accumulation of pyruvate and lactate in a nutrient-deprived environment has been suggested to promote HIF-1α stabilization through the plasminogen activator inhibitor-1 (PAI-1) and the lactate transporter monocarboxylate transporter 1 (MCT1). Under normoxic conditions, the increase in glutamine consumption leads to an increase in intracellular ammonia concentration, which results in stabilizing HIF-1α in different tumor cell lines [47,48]. A previous report identified the role of mTORC1 under normoxic conditions in activating HIF-1α by regulating the initiation factor 4E-binding protein 1 (4E-BP1) and ribosomal protein S6 kinase-1 (S6K1) [49]. mTORC signaling is involved in the adaptive cell response against nutrition deprivation. The PTEN/PI3K/AKT signaling axis was shown to increase HIF-2α expression and stability via an mTORC2-dependent mechanism [50,51].

The regulation of HIF-1α and HIF-2α is also significantly influenced by epigenetic mechanisms in the absence of a hypoxic environment. Histone deacetylases (HDACs) and sirtuins (SIRTs) can regulate HIF-1α by either enhancing its stability or promoting further degradation. Demethylases such as JMJD1A and LSD1 increase the transcriptional activity of HIF-1α by demethylating histones or HIF-1α itself [52,53]. ARD1 acetylates HIF-1α, tagging it for ubiquitination and promoting its breakdown [54,55]. Acetyltransferases such as TIP60 and PCAF enhance HIF-1α stability and transcriptional activity by acetylating specific lysine residues on HIF-1α or histones associated with its target genes [56]. Additionally, it was shown that SIRT1 deacetylates HIF1α’s lysine residues, leading to repression of HIF-1α transcription [19]. SET7/9 and PRMT1 are methyltransferases that inhibit HIF-1α by reducing its mRNA transcription or stability [57,58], while its translation is supported by PRMT5. SET7/9 methylates HIF-2α, which inhibits the transcription of its target genes [59]. SIRT1 enhances HIF-2α transcriptional activity through deacetylation, promoting the expression of HIF-2α-dependent genes [60]. Additionally, DNMT3a, a DNA methyltransferase, was shown to regulate HIF-2α expression by modifying the DNA methylation status of its target genes, influencing their transcription [61].

## 5. Differential Expression Patterns and Functions of HIF-1α and HIF-2α

The expression of many HIF target genes is controlled by HIF isoforms in a cell type-specific manner. HIF-1α is found in most adult mammalian tissues, while HIF-2α is mainly expressed in specific cell types such as the endothelium, hepatocytes, intestinal epithelial cells, pancreatic cells, and alveolar epithelial cells [62,63,64,65]. In vivo studies involving knockout mice have shown that both HIF-α subunits are essential for development and serve non-redundant functions. HIF-1α knockout mice demonstrated mid-gestation mortality (at stage E11) due to significant cardiovascular anomalies and decreased erythropoiesis [66]. This indicates that HIF-1α is crucial for the early development of the cardiovascular system [67]. On the other hand, HIF-2α knockout mice died at a later stage (E13.5) due to impaired vascular remodeling, reduced catecholamine homeostasis, and lung immaturity [68,69]. Intracellular HIF-1α and HIF-2α are accumulated differently based on the levels and duration of hypoxia. The HIF-1α isoform primarily accumulates at lower oxygen levels (0–2% O_2_) in cancer cell lines, while the increased level of stabilized HIF-2α is maintained during moderate hypoxia levels (2–5% O_2_) [70]. The two isoforms have also shown a distinct temporal pattern; HIF-1α is primarily stabilized in the acute response to hypoxia, activating genes promoting immediate cell responses such as metabolic adjustments and survival signals. On the other hand, HIF-2α seems to control genes contributing to long-term cellular and systemic adaptative responses to chronic hypoxia such as erythropoiesis [71].

Although HIF-1α and HIF-2α are able to bind the same consensus sequence, their target genes can vary depending on the cellular and molecular context. The effect of HIF-1α and HIF-2α subunits on their target gene regulation was unraveled by high-resolution ChiP-seq studies, which identified over 500 high-affinity sites of HIF binding throughout the genome. These binding sites are frequently found close to genes that are essential to vital functions such as metabolism, cell survival, and growth [72,73]. Moreover, the binding sites of HIF-1α and HIF-2α could be mutual between them, which indicates that their cooperative actions on some of their target genes could coordinate the cell’s response against hypoxia, while other binding sites were identified as unique for each HIF isoform, suggesting their distinct action for some target genes. HIF target gene specificity can be explained by their interaction with the NTAD region in HIF-α isoforms. The replacement of the NTAD of HIF-2α with the NTAD of HIF-1α established the functional specificity of HIF-1α for their gene targets [74].

HIF-1α and HIF-2α share the regulation of several important target genes involved in biological functions, such as angiogenesis, glucose transport, and cellular homeostasis, enabling the cell to survive in oxygen-deprived environments. Despite their intersecting functions, each isoform can control distinctive target genes based on their unique roles in different hypoxic contexts. HIF-1α predominantly governs genes related to rapid adaptation to hypoxia, focusing on immediate responses such as metabolism and angiogenesis. In contrast, HIF-2α plays a more significant role in regulating genes involved in long-term responses to chronic hypoxia, such as erythropoiesis and vascular remodeling. Examples of common and unique target genes for HIF-1α and HIF-2α are summarized in (Table 1).

## 6. The Differential Role of HIF-1α and HIF-2α in the Metabolic Switch from Oxidative Phosphorylation to Glycolysis

In response to reduced oxygen availability, cells undergo a metabolic shift from oxidative phosphorylation to glycolytic metabolism. This fundamental adaptation is crucial for tumor cell survival under the existing hypoxic microenvironment of some tumor types [105,106]. By converting to glycolysis, cells produce ATP and NADPH, two compounds necessary for cell growth and proliferation, even though glycolysis produces less ATP per glucose molecule than mitochondrial oxidation [107]. To coordinate the most efficient use of oxygen, HIFs activate a network of genes that rewire cellular metabolism, shifting energy production away from oxygen-intensive oxidative phosphorylation and toward anaerobic glycolysis [108].

Both HIF-1α and HIF-2α contribute to metabolic reprogramming by regulating some common target genes implicated in metabolism, such as glucose transporters [109]. However, they serve distinct roles in regulating energy metabolism under hypoxic conditions. HIF-1α is more dominant in controlling glycolysis, rapidly initiating the adaptive response to acute hypoxia by upregulating genes that enhance glycolysis and suppress oxidative phosphorylation [110]. This involves the direct activation of transcription for essential enzymes in the glycolytic pathway, such as hexokinase, phosphofructokinase, aldolase, pyruvate kinase M2 (PKM2), and lactate dehydrogenase (LDH), facilitating the conversion of glucose to lactate [2,80]. The lactate produced during glycolysis is a substrate for the biosynthesis of macromolecules, including lipids, proteins, and nucleic acids, vital for proliferation and survival. Additionally, HIF-1α enhances glucose uptake by increasing the expression of glucose transporters, notably GLUT1 and GLUT3, ensuring an abundant supply of substrate for glycolysis [111,112].

Beyond its function in glycolysis, HIF-1α regulates additional metabolic adaptations that optimize energy production and mitigate oxidative stress. It modulates mitochondrial function by inducing the expression of BNIP3 and BNIP3L, proteins that promote mitophagy, which is the selective degradation of damaged mitochondria [113]; removing dysfunctional mitochondria reduces overall mitochondrial mass and oxygen consumption, limiting the production of reactive oxygen species (ROS) and protecting cells from oxidative damage. This process is critical for maintaining cellular health and function [114,115]. Furthermore, HIF-1α suppresses mitochondrial biogenesis by inducing the expression of transcriptional repressors such as MXI1. This mechanism antagonizes the activity of c-Myc, resulting in a reduction in mitochondrial oxygen demand. HIF-2α is primarily involved in the metabolic adaptation to chronic hypoxia and has a wider influence on energy homeostasis and biosynthetic pathways [116].

Unlike HIF-1α, HIF-2α does not strongly induce glycolytic enzymes but instead regulates genes involved in lipid storage and metabolism, amino acid metabolism, and oxidative phosphorylation [117,118]. Under prolonged hypoxia, HIF-2α can decrease fatty acid metabolism by directly upregulating genes involved in lipid storage such as the adipose differentiation-related protein (ADFP/PLIN2) and by repressing fatty acid oxidation of the indirect regulation of key enzymes, including acyl-CoA synthetase long-chain family member (ASCL1), carnitine palmitoyltransferase 1 (CPT1), and peroxisome proliferator-activated receptor alpha (PPARα). HIF-2α decreases lipid synthesis by suppressing critical lipogenic transcription factors and enzymes such as sterol regulatory element-binding protein 1c (SREBP-1c) and fatty acid synthase (FASN) [119,120]. Despite HIF-1α being the most expressed HIF isoform in cells, and the fact that its stability is correlated with the hypoxia level, HIF-2α is less likely to degrade under prolonged hypoxia and pathological hypoxic conditions. For instance, VHL mutations result in the accumulation of HIF-2α in renal clear cell carcinoma. In this cancer type, the activation of HIF signaling is associated with the accumulation of both glycogen and lipid droplets in renal clear cell carcinoma [121]. In metabolic disorders, the deletion of HIF-1α enhances lipid accumulation in the animal model of alcoholic fatty liver via the induction of SREBP-1c [122]. In contrast, HIF-2α activation leads to the development of severe hepatic steatosis in adult mice. This was associated with a reduction in fatty acid oxidation and increased lipid storage levels [117]. It seems that under HIF-1α dominance, cells favor ATP production through glycolysis and limiting lipid synthesis and fatty acid beta-oxidation.

**Figure 3:**
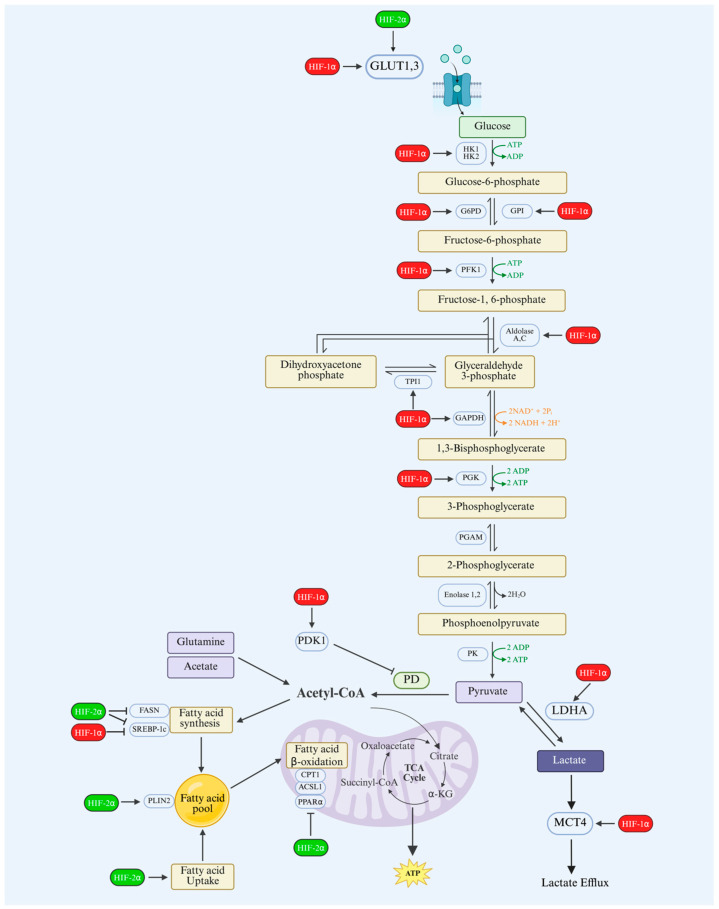
The roles of HIF-1α and HIF-2α in the regulation of glucose and lipid metabolism. During hypoxia, HIF-1α promotes glycolysis by inducing glycolytic enzymes, including glucose transporters (GLUTs), hexokinase 1 (HK1), hexokinase 2 (HK2), glucose-6-phosphate dehydrogenase (G6PD), glucose-6-phosphate isomerase (GPI), phosphofructokinase 1 (PFK1), aldolase A (ALDOA), aldolase C (ALDOC), triosephosphate isomerase 1 (TPI1), glyceraldehyde-3-phosphate dehydrogenase (GAPDH), phosphoglycerate kinase 1 (PGK1), enolase 1 (ENO1), enolase 2 (ENO2), and lactate dehydrogenase A (LDHA), enhances lactate efflux via monocarboxylate transporter 4 (MCT4), and suppresses mitochondrial oxidative metabolism via pyruvate dehydrogenase kinase 1 (PDK1)-mediated inhibition of pyruvate dehydrogenase (PD). On the other hand, HIF-2α enhances lipid storage by inducing perilipin 2 (PLIN2) and suppressing fatty acid β-oxidation indirectly via the downregulation of carnitine palmitoyltransferase 1 (CPT1), acyl-CoA synthetase long-chain family member 1 (ACSL1), and peroxisome proliferator-activated receptor alpha (PPARα), as well as fatty acid synthesis by suppressing sterol regulatory element-binding protein 1c (SREBP1C) and fatty acid synthase (FASN). The figure was created with BioRender.com.

Conversely, since HIF-2α does not induce glycolytic enzymes like HIF-1α, pyruvate dehydrogenase remains active under HIF-2α stabilization during the chronic phase of hypoxia [118]. Therefore, the content of pyruvate would not be significantly affected, and subsequently the conversion rate to acetyl-CoA produced by TCA cycle, which is one important substrate for fatty acid synthesis, would also not be altered. In hypoxic cancer cells, alternative sources of Acetyl-CoA are available by inducing the conversion of glutamine to α-ketoglutarate and its subsequent production of citrate, which is converted into Acetyl CoA. Also, most cancer cells rely on acetate as another alterative substrate for Acetyl CoA production [123,124,125]. In addition, it has been shown that HIF-2α can increase the expression of lipid transporters such as CD36, leading to enhanced uptake of circulating fatty acids into cells [126]. Taken together, cellular lipid storage tends to be increased if HIF-2α is activated (Figure 3).

By mediating these metabolic pathways, HIF-1α and HIF-2α provide cells with versatile survival strategies under hypoxic conditions. Targeting these factors or their downstream metabolic effectors represents a promising therapeutic approach for disrupting adaptation to hypoxia in several diseases such as cancer, diabetes, and liver lipid disorders.

## 7. The Interrelation Between HIF-1α and HIF-2α (The HIF Switch)

**Figure 4:**
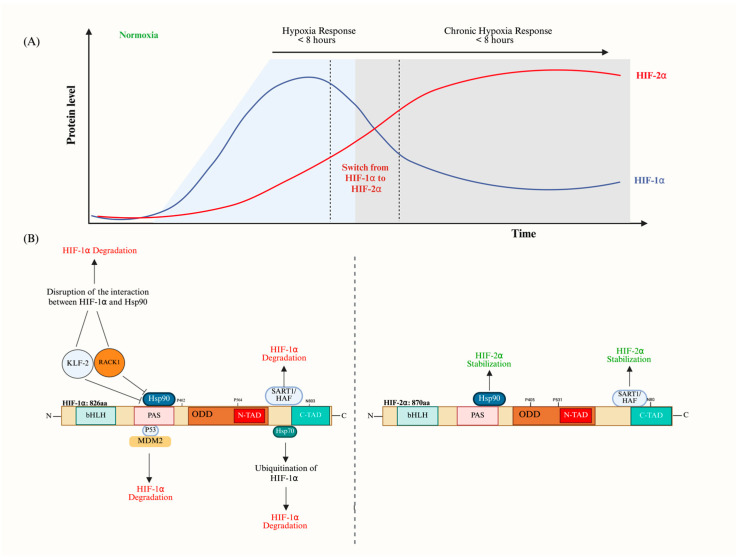
The transition from HIF-1α to HIF-2α stabilization. (**A**) The HIF switch’s temporal dynamics in response to protracted hypoxia. The HIF-1α level rapidly builds up within the first few hours. A reduction in the HIF-1α level starts at the 8 h mark as HIF-2α levels start to rise and last for more than 24 h. (**B**) Multiple players acting on HIF-1α causing its subsequent degradation, including KLF2, RACK1, MDM2, and HAF. HSP90 and HAF work on the stabilization of HIF-2α. The figure was created with BioRender.com.

The HIF switch is a critical adaptive mechanism that allows cells to accordingly interchange their responses to varying levels and durations of oxygen deprivation [15]. This switch involves a dynamic transition between HIF-1α and HIF-2α in the same context of the cell type and environment [71]. The accumulation of HIF-1α is characteristic of the onset of hypoxia, promoting initial adaptive responses such as glycolysis and angiogenesis to increase oxygen availability. The HIF switch is thought to be controlled by the fact that HIF-1α causes a transition to the glycolytic pathways, which reactivates PHD activity and leads to the degradation of HIF-1α [127]. HIF-1α mRNA has a significantly shorter half-life than EPAS1 mRNA during chronic hypoxia, suggesting that differences in mRNA stability contribute to the HIF transition. The increase in hypoxia necessitates further adjustments at the metabolic and vascular levels to prevent extensive oxidative damage [128]. This transition from HIF-1α to HIF-2α highlights the intricate molecular mechanisms driving the shift from HIF-1α to HIF-2α’s dominance, serving as an adaptive technique to chronic hypoxia and revealing the complex interaction of regulatory elements controlling the cellular response to oxygen insufficiency [64] (Figure 4A).

Key factors involved in this transition include SART1/HAF, which selectively destabilizes HIF-1α while enhancing HIF-2α stability and transactivation. SART1/HAF, the human homolog of the murine hypoxia-associated factor, binds and destabilizes HIF-1α in a pVHL-independent, proteasome-dependent manner under both normoxic and hypoxic conditions, without affecting HIF-2α levels. Instead, HAF enhances HIF-2α transcriptional activity by binding to its specific C-terminal domain, effectively converting cells from an HIF-1α to an HIF-2α transcriptional program [129,130]. Additionally, HIF-1α stabilization is influenced by its interaction with Heat Shock Protein 90 (Hsp90), which is disrupted by the Receptor for Activated C Kinase 1 (RACK1), allowing HIF-2α to build up [131]. HSP70 also plays a role in this transition by interacting with CHIP, which causes the ubiquitination and degradation of HIF-1α while leaving HIF-2α relatively unaffected [132].

Kruppel-like Factor 2 (KLF2), a crucial component in endothelial cell growth and healthy vessel development, further broadens the regulatory framework of endothelial cells by disturbing the interaction between HIF-1α and Hsp90 under hypoxic conditions, leading to selective degradation of HIF-1α and altering the course of the HIF switch [133]. Mouse Double Minute 2 Homolog (MDM2) is another significant regulator, promoting the ubiquitination and proteasomal destruction of HIF-1α under normoxic conditions in conjunction with p53 [134] (Figure 4B).

The role of microRNAs (miRNAs) in the HIF switch mechanism adds another layer of complexity. These noncoding RNA molecules regulate protein levels by binding to the 3′UTR of mRNAs, influencing both HIF-1α and HIF-2α stability [135]. For instance, miR-424 promotes HIF-1α stability by decreasing the expression of Cullin-2, a scaffolding protein necessary to assemble the HIF E3 ubiquitin ligase complex [136]. While many miRNAs have been identified to bind to HIF-1α mRNA and cause its degradation, very few miRNAs have been demonstrated to interact with HIF-2α mRNA [137]. Indeed, HIF-2α mRNA seems to be resistant to miRNA-dependent degradation. In addition, chronic hypoxia compromises the biogenesis of miRNA, affecting the post-transcriptional regulation of HIF-2α [138,139].

When HIF is stabilized in tissue following hypoxic insult, the subsequent adaptative processes such as the formation of new blood vessels can restore the oxygen level supply. Both HIF isoforms are degraded very quickly upon this reoxygenation period. HIF-1α DNA-binding capacity starts to diminish after only 2 min of reoxygenation [140]. However, in some aggressive cancers, HIF-1α may remain stabilized in the presence of oxygen [141]. Even though HIF-α stabilization under a normoxic environment does not lead to cell death, it costs more energy to maintain the adaptive processes, including activating energetically inefficient glycolysis. Indeed, it was found that HIF-α switches from a facultative activation pattern to a constitutive pattern in some tumor types, which causes sustained aggressive behavior including uncontrolled proliferation and excessive angiogenesis [142,143]. The processes mentioned above contribute to the HIF switch and heavily rely on stabilizing/destabilizing the α subunits, leading to the development of pathogenesis if not properly controlled in a timely manner.

## 8. Role of HIF-1α and HIF-2α in Angiogenesis

Blood vessels are the primary transporters that carry oxygen and nutrients to all body tissues to fulfil cell metabolism needs. Many factors can lead to decreased oxygen availability for adult tissues, including feeding of the artery blockade, tumor growth, or increases in metabolic demands, thus altering cell metabolism. Both hypoxia and the consequent metabolic stress can trigger an adaptive response, growing new blood vessels for maintaining tissue homeostasis. HIF signaling plays a critical role in the oxygen-sensing mechanisms that regulate the growth of the new blood vessels.

In the embryo, the low oxygen level stimulates the differentiation of progenitors into hemangioblasts [144]. The HIF-1α and HIF-1β subunits work together to regulate the early stages of de novo blood vessel formation (vasculogenesis) via the paracrine effect of pro-angiogenic growth factors such as vascular endothelial growth factor (VEGF) secreted from hematopoietic cells. Genetic studies show evidence of severe structural abnormalities of blood vessels during placental development in mouse embryos deficient in either HIF-1α or HIF-1β [145]. HIF-2α loss results in abnormal vessel remodeling in the embryo and yolk sac, which causes swelling and hemorrhage in the vessels [146]. In a similar manner, in adult bone marrow, when vascular progenitors are exposed to low O_2_ tensions, HIF-1α is activated, promoting their transition into more differentiated endothelial cells that express mature endothelial markers: CD31, VEGFR2, and endothelial NO synthase (eNOS) [147].

Sprouting angiogenesis is the most common cellular mechanism of forming new vessels. It is initiated by a selection of quiescent endothelial cells that behave as tip cells, followed by stalk cells. Endothelial tip cells are invasive and guided by vascular growth factor gradients such as VEGF. VEGFR2 expressed on endothelial tip cells is activated and induces migration. VEGF and other vascular growth factors, such as fibroblast growth factor (FGF), are secreted by neighboring non-vascular cells, such as fibroblasts, immune cells, and tumor cells. HIF-1α effectively stimulates the expression of vascular endothelial growth factor A (VEGF-A) in many cell types [148]. Lactate is a metabolic end-product of glycolysis that acts as a metabolic fuel for oxidative cells and a signaling molecule in several cell types [149]. It has been shown that it can inhibit PHD2 activity, leading to stabilizing HIF-1α in normoxic oxidative tumor cells. This HIF-1α activation upregulates VEGF expression and other proangiogenic cytokines [150]. In renal cancer cells, HIF-2α can compensate for HIF-1α deficiency by sustaining VEGF expression [151]. The interaction between VEGF and Notch signaling has a key role in the process of tip cell selection. Tip cells express higher levels of Delta-like canonical notch ligand 4 (Dll4) with low notch signaling activity compared with stalk cells. Hypoxia induces the Notch ligand Dll4 in endothelial cells [152]. Both HIF-1α and HIF-2α are able to upregulate the activation of Dll4-mediated Notch signaling and can also directly activate promoter of the Notch downstream transcription factor Hey1. Moreover, HIF-1α activates the C-X-C motif chemokine receptor 4 (CXCR4) axis [153]. Additionally, HIF-1α selectively upregulates neuropilin-1 (NRP1), which acts as a co-receptor for VEGF. This interaction enhances VEGFR-2 signaling in tip cells, facilitating their activation and promoting directional migration [154].

HIF-1α enhances the expression of glycolytic enzymes, including hexokinase-2 (HK2) and lactate dehydrogenase A (LDHA) in stalk cells. These enzymes facilitate stalk cell proliferation under hypoxic conditions, promoting the elongation of blood vessels. This metabolic shift allows stalk cells to continue growing and extending the developing vessel even in low-oxygen environments [155]. In contrast, it has been recently reported that HIF-2α accumulation in PHD2-deficient ECs may severely restrict vascular growth in part by upregulating DLL4 expression and the activation of NOTCH signaling. Earlier studies showed that PHD2 haplodeficiency normalizes the leaky tumor vasculature primarily through the regulation of HIF-2α [156]. In contrast, the endothelial cell-specific knockout of HIF-2α by using VE-cadherin Cre-recombinase mice exhibited vascular leakage in the lungs and fat, further supporting the notion that HIF-2α promotes vessel maturation in ECs [157]. HIF-2α also induces the expression of angiopoietin-1 (ANG-1), resulting in the detachment of pericytes from endothelial cells during the initiation process of angiogenesis [158]. This detachment is critical for vascular remodeling, as it permits the formation of new blood vessels in response to hypoxic conditions [159]. HIF-2α plays a critical role in strengthening endothelial junctions by upregulating the expression of VE-cadherin, which is essential for stabilizing these junctions. This stabilization is imperative for preventing the collapse of junctional structures into the lumen, thereby facilitating the formation of robust intraluminal structures that are integral to vascular remodeling processes [160]. Taken together, it seems that HIF-2α expression in ECs is specifically required for vascular integrity and remodeling.

Each HIF-α isoform plays important and distinct roles in adult angiogenesis. HIF-1α and HIF-2α can partly compensate or complement each other. HIF-2α complements HIF-1α by sustaining prolonged expression of VEGF through the upregulation of Ang-1 [158]. This enhancement in VEGFR-2 signaling supports the angiogenic response, ensuring that the newly formed sprouts receive the necessary signals for continued growth and stabilization. The vessel maturation and stabilization phases encompass the establishment of the basement membrane alongside the recruitment of mural cells, such as pericytes, serving a critical function in preserving vascular integrity and promoting interactions between endothelial cells and the surrounding extracellular matrix. HIF-1α contributes to this process by regulating the expression of key factors, such as platelet-derived growth factor (PDGF-B) [161]. PDGF-B facilitates vessel stabilization by recruiting pericytes, which wrap around endothelial cells to provide mechanical support [162]. HIF-1α is involved in preserving tight junctions through the regulation of proteins such as claudin-5 during the initial development of the lumen in newly formed blood vessels [163]. HIF-2α complements the function of HIF-1α by upregulating elastin and fibronectin, thereby contributing to the stability of the extracellular matrix (ECM) surrounding the intraluminal pillars [164]. This stability is essential for maintaining the structural integrity of the ECM as the sprouts expand, supporting the expansion and maturation of these new intraluminal structures within the vascular lumen.

**Figure 5:**
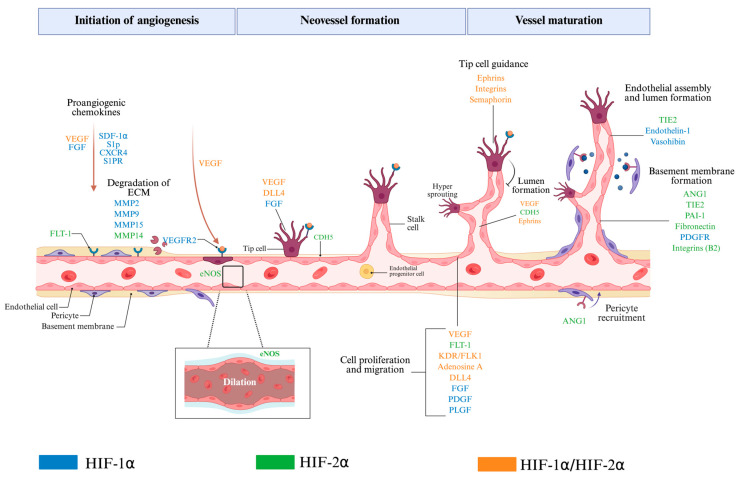
HIF-1α and HIF-2α regulation of angiogenesis from initiation to vessel maturation. HIF-1α and HIF-2α play complementary roles in driving angiogenesis. While they share some common gene targets, each one activates specific genes at different stages, working together to guide blood vessel formation. Under hypoxic conditions, they take part in multiple steps of angiogenesis including increasing permeability, degrading the surrounding matrix, guiding tip cell movement, stimulating endothelial cell growth, forming the vessel lumen, building the basement membrane, interacting with pericytes, and stabilizing the vessel. Gene targets colored in red are regulated by HIF-1α, green is for HIF-2α, and orange is for genes regulated by both. The figure was created with BioRender.com.

Other examples of such complementary actions between HIF-1α and HIF-2α include their coordinated effect on basement membrane degradation. This process is important to enable endothelial cells to migrate through the tissue and initiate the formation of new branches [137]. HIF-1α enhances the expression of matrix metalloproteinases (MMP-2 and MMP-9), which are essential enzymes responsible for breaking down extracellular matrix (ECM) components, including collagen and fibronectin. Furthermore, HIF-1α stimulates the activation of urokinase-type plasminogen activator (uPA) and plasminogen activator inhibitor-1 (PAI-1), both of which further facilitate ECM degradation and promote cellular migration. While HIF-2α is involved in regulating multiple genes, including the adenosine A2A receptor, MMP-14 and MMP-17, PAI-1, EPO, fibronectin, and integrins α9, and β2 [165,166,167,168,169,170] (Figure 5).

## 9. Conclusions

Even though HIF-1α and HIF-2α isoforms share high similarity in their structure and target genes, much evidence shows that they have distinct functional roles in the context of cell metabolism and angiogenesis. For instance, HIF-1α is involved in the adaptive acute response to hypoxia by regulating glycolysis and the early steps of angiogenesis, while HIF-2α is involved in sustaining the follow-up response to hypoxia by stabilizing the cellular metabolic state, increasing EPO production, and promoting vascular remodeling. In that perspective, many small molecules have been developed to selectively inhibit the HIF-2α isoform in tumors with proven elevated HIF-2α levels [171]. These inhibitors block the interaction between HIF2α and ARNT, stopping HIF2α-induced transcription [172]. For instance, Belzutifan has been approved clinically as a second-generation specific HIF-2α inhibitor for patients with Von Hippel–Lindau (VHL) syndrome to treat associated clear cell renal carcinoma and central nervous system hemangioblastomas [173]. Heterozygous deficiency of PHD2 in mice normalized tumor microvessels and improved tumor perfusion. Tumor vessel normalization resulted in a reduction in tumor cell invasion and metastasis [156]. These findings suggest that HIF-2α is involved in endothelial normalization since HIF-2α binds to hypoxia-responsive elements in the VE-cadherin and Flt1 promoter in endothelial cells [174]. An early study demonstrated that transgenic mice constitutively expressing active HIF-1α in the skin exhibit skin vascularity with an elevation of VEGF expression [175]. HIF-1α and HIF-2α adenovirus-mediated gene transfers increased capillary diameter in ischemic skeletal muscles, and in particular, HIF-2α gene transfer showed stronger capillary growth in muscles [176]. Targeting prolyl hydroxylases (PHDs) to enhance HIF activity presents a promising therapeutic strategy to protect tissues against ischemia. However, current small-molecule PHD inhibitors inhibit all three PHD enzymes and induce erythropoietin (EPO) expression. Many PHD inhibitor compounds are currently approved for clinical use to manage anemia in patients with chronic kidney disease in Japan and China [177]. It has been shown that the in vivo stimulation of EPO expression and erythropoiesis is mainly due to the activation of HIF-2α [178]. Indeed, gain-of-function mutations introduced to HIF-2α are sufficient to overexpress EPO in the kidney and liver and enhance erythrocytosis [179]. Excessive erythropoiesis can increase the risk of cardiovascular disease and mortality. Specific inhibition of PHD2 using an inducible short hairpin RNA (shRNA) interference gene knockdown showed cardiac protection against ischemia insult in mice without increased erythrocytosis [180]. The HIF switch, which represents the dynamic interrelationship between both isoforms, is an essential mechanism to prevent excessive metabolic stress and maintain vascular homeostasis. The impairment of this HIF switch mechanism could contribute to the development of various pathological conditions, including cancer, ischemic diseases, and inflammatory disorders. A better understanding of the interrelationship between both HIF isoforms would have significant implications for implementing future therapeutic strategies to restore the physiologic HIF switch.

## Figures and Tables

**Table 1:** Comparative expression and target genes for HIF-1α and HIF-2α in hypoxia.

	HIF-1α	HIF-2α
**Tissue expression**	Ubiquitous	Endothelium, hepatocytes, intestinal epithelial cells, pancreatic cells, and alveolar epithelial cells
**Timing of expression**	Acute hypoxia exposure (2–24 h)	Chronic hypoxia exposure (48–72 h)
**Unique target genes**	NRF2 [75], FGF2 [76], ALDH1A [77], CAIX [78], ALDOA [79], ALDOC [80], TPI1 [81], PGK1, ENO A1, PKM2, LDHA, PFK [82], HK1, HK2, GPI, PDK1 [83], iNOS [84], BNIP3 [85], CXCR4 [86], and NRP1 [87]	CCND1 [88], MCT1 [89], VEGFC [90], BAK [91], ABL2 [92], FSTL [93], EPO [94], ANG-2 [95], TGFα [96], FLT1 [97], PLIN2 [88], and OCT4 [98]
**Common target genes**	VEGFA, IL-6 [99,100], ADM [101], NDRG1 [102], CAXII, GLUT1, ADRP [63], MMPs [103], and DLL4 [104]	

## Data Availability

Not applicable.
